# TJ-41 Induces Apoptosis and Potentiates the Apoptotic Effects of 5-FU in Breast Cancer Cell Lines

**DOI:** 10.1155/2009/895381

**Published:** 2009-08-20

**Authors:** Suresh Volate, Robert Hudson, Desuo Wang, Stephanie Muga, Michael Wargovich

**Affiliations:** ^1^Division of Basic Research, South Carolina Cancer Center, 14 Medical Park, Suite 500, Columbia, SC 29203, USA; ^2^Division of Cell and Molecular Pharmacology, Hollings Cancer Center, 86 Jonathan Lucas St., HO712H Charleston, SC 29425, USA

## Abstract

Recent studies suggest that TJ-41, a herbal drug, possesses chemotherapeutic effects. Accordingly, this study was undertaken to investigate the anticarcinogenic effects of TJ-41 on human breast cancer cells lines. TJ-41 inhibited the proliferation of human breast cancer cell lines dose dependently. Flow cytometric analysis showed that this decrease in DNA synthesis is to be associated with induction of apoptosis. In both cell lines, apoptosis was abolished by caspase-9 inhibitor Z-LEHD-fmk but was weakly inhibited by caspase-8 inhibitor Z-IETD-fmk, indicating that caspase-9 activation was involved in TJ-41 induced apoptosis. Additionally, TJ-41 stimulated phosphorylation of c-Jun NH2-terminal kinase (JNK) and pretreatment of breast cancer cells with JNK inhibitor SP600125 completely abolished TJ-41 induced apoptosis. Our data also demonstrate that combined treatment of TJ-41 and 5-FU significantly potentiates the apoptotic effects of 5-FU in both breast cancer cell lines. Taken together, these data suggest that TJ-41 might provide a novel chemotherapeutic treatment for breast cancer.

## 1. Introduction

 Despite centuries of theoretical meandering and scientific inquiry, breast cancer (BrCa) continues to represent a burgeoning medical problem [1]. According to the World Health Organization, more than 1.2 million people will be diagnosed with breast cancer each year worldwide. The average lifetime risk of BrCa for newborn U.S. females is 12%, and this risk is much higher in patients with certain risk factors such as early menarche, nullparity, and late menopause [2]. The American Cancer Society estimates that in this year 216 000 new cases of breast cancer will be diagnosed and that roughly 40 000 women will die of this disease. A major effort to reduce breast cancer mortality and morbidity is focused on development of better breast cancer treatments. 

In the past few years, it has become apparent that cancer patients are actively seeking complementary/alternative approaches to serve either adjunctively with allopathic chemotherapy or singly as a replacement for primary therapy. In a recent study assessing the prevalence of complementary and alternative medicine (CAM) in a comprehensive cancer center, 83.3% of patients had used at least 1 CAM approach and 62.6% had used vitamins and herbs [3]. A number of recent studies support the efficacy and safety of some herbal formulas. In addition, these studies have shown that these remedies worked effectively in some instances in which conventional Western therapies failed or proved to be insufficient to provide a palliative cure. For example, a randomized, nonblind, controlled clinical trial conducted in Japan showed that sho-saiko-to, an extract of seven Chinese herbs, helps preventing liver cancer in patients with cirrhosis [4].

The use of Chinese herbal drugs, which have an extensive history of use (3000 yrs in China) has now been increasing in recent years because of their safety and few side effects [5]. Many herbal formulas are believed to have tonic effects to body function. Among them, TJ-41, named Bu-Zhong-Yi- Qi-Tang in China and Hochu-Ekki-To in Japan, is considered to be the best at nourishing the vital energy-Qi [6] and antagonizing the adverse effects caused by chemotherapy [7–9]. Cell cultures [10], animal studies [11], and clinical trials have demonstrated that TJ-41 formula may inhibit tumor cell proliferation, enhance immune activity of lymphocytes, and improve cancer patients' well being [12]. Accordingly, the first objective of this study was to characterize the effects of TJ-41 on hormone sensitive and insensitive breast cancer cell lines (MCF-7 and MDA-MB-231, resp.).

5-fluorouracil (5-FU) is a DNA damaging drug that is commonly used as a chemotherapeutic agent that inhibits cancer growth by initiating apoptosis. 5-FU initiates apoptosis by targeting thymidylate synthase (TS), and by direct incorporation of 5-FU metabolites into DNA and RNA [13]. This drug has been commonly used to treat various types of cancer including breast, head, stomach, and colon cancer. Ideally, chemotherapeutic drugs should specifically target only cancerous cells by inducing cytotoxic or cytostatic effects, thereby decreasing the tumor growth without affecting normal cells. The effectiveness of traditional chemotherapy has suffered due to the lack of specificity, rapid drug metabolism, and both intrinsic and acquired drug resistance as well as induction of side effects due to high dosage. This produces a transient decline in quality of life of the patients. These problems could be overcome by a low-dose treatment of drugs in combination with other natural compounds/drugs, which could enhance the antiproliferative effects. Therefore, the second objective of the present study was to explore the combined effect of 5-FU and TJ-41 on MCF-7 and MDA-MB-231 cell lines. 

## 2. Methods and Materials

### 2.1. Preparation of TJ-41

TJ-41 was purchased from Xi’an National Chinese Herbal Cooperation and consists of crude standardized extract from ten herbs. The plant materials and their specific ratio (in grams) as they are described in the company literature are *Astragalus membranaceus* (10.0 g), *Panax ginseng* (10.0 g), *Atractylodes lancea rhizome* (10.0 g), *Angelicae sinensis* (7.5 g), *Radix bupleuri* root (5.0 g), *Glycyrrhiza glabra* root (3.8 g), *Rhizome cimicifuga* (2.5 g), *Ziziphus zizyphus* (5.0 g), *Citrus unshiu* peel (5.0 g), and *Zingiber officinale* (1.3 g). Seventy five grams of TJ-41 were poured together for extraction in a glass container and soaked for 24 hours with 2000 mL tap-water in refrigerator. The next day, the mixture was boiled for more than 35 minutes halving the original volume. The decoction was filtered through a colander while hot, and the volume adjusted to 1000 mL with water after cooling. The adjusted decoction was centrifuged (8.000 rpm for 10 minutes) and the supernatant was sterilized by sequentially passing it through 0.45-*μ*m and 0.22-*μ*m filter. The stock solution of TJ-41 (75 mg/mL) was aliquoted into 50 mL centrifuge tube and stored at −20°C.

### 2.2. Cell Culture

 The human breast adenocarcinoma ER+ (MCF-7) and ER− (MDA-MB-231) cell lines were obtained from ATCC. MCF-7 cell line (*estrogen receptor positive*) was derived from breast cancer pleural effusion and MDA-MB 231 (*estrogen receptor negative*) was derived from the lung metastases of another breast cancer patient. Cell lines were grown in monolayer culture in DMEM (Dulbecco's Modified Eagle's Medium) supplemented with 10% (v/v) fetal calf serum, and 1% (v/v) antibiotic-antimycotic agent. Cells were grown to confluence at 37°C in a humidified atmosphere containing 5% CO_2_ in air and were passaged weekly using 0.25% trypsin. The experiments were performed with cells in the logarithmic phase of growth. 

### 2.3. Proliferation Assay and Reversibility Study

 Human breast cancer (BrCa) cell lines were seeded at a density of 5 × 10^4^/well in 24-well tissue culture dishes and allowed to attach overnight. To synchronize the cell cycle, BrCa cells were incubated in serum-free medium for 24 hours. Triplicate samples of growing cells were treated with TJ-41 for time and concentrations as indicated in the experiments. During the last 6 hours of treatment, cells were pulsed with [^3^H]-Thymidine (1 *μ*Ci mL^−1^) and processed as reported by Brandi et al. [14]. The results are expressed as mean percentage of [^3^H]-Thymidine incorporation from triplicate wells as compared with control untreated samples. For the reversibility study, both MCF-7 and MDA-MB-231 BrCa cells were treated exactly as described above except, after synchronization of cell cycle they were incubated with 375 *μ*g/mL of TJ-41. After 2 days, the medium was removed and the cells were further incubated in a fresh drug-free medium for 2 more days. During the experiment, medium was replaced with a fresh drug-containing or drug-free medium every 24 hours. At the end of incubation, cell proliferation was assessed by incorporation of [^3^H]-Thymidine as described above. For this assay, each concentration (each time) was performed in triplicates wells, and in at least 3 independent experiments.

### 2.4. Analysis of the DNA Content by Flow Cytometry (Hypoploid Cells)

MCF-7 and MDA-MB-231 cells were seeded at a density of 5 × 10^5^/well in six-well culture dishes. To synchronize the cell cycle, BrCa cells were incubated in serum-free medium for 24 hours. The cells were then treated with or without 75 *μ*g/mL TJ-41 and 0.5 *μ*M 5-FU (Sigma Aldrich Co. St. Louis, Mo, USA) and harvested after 48 hours by trypsinization. The cells were centrifuged at 2000 rpm for 5 minutes, washed with phosphate-buffered saline (PBS), fixed with 70% ethanol, and then subjected to flow cytometric analysis (Epiccs XL-MCL, Beckman Coulter) after propidium iodide labeling. This assay was performed at least 3 independent times for each treatment group. For each experimental condition a minimum of 3 × 10^4^ cells was analyzed. Data were acquired using Expo32Acquision software (Applied Cytomety System, Beckman Coulter, Miami, Fla). The percentage of cells with subdiploid DNA was quantified by the Expo 32 ADC (Applied Cytomety System, Beckman Coulter, Miami, Fla) software by plotting intensity of fluorescence versus number of cells. The percentage of cells located to the left of the G_1_ peak, diagnostic of hypodiploid cells that have lost DNA, was taken as the percentage of apoptotic cells [15]. For the experiments that use inhibitors, cells were pretreated with 10 *μ*M of JNK inhibitor SP600125, or caspase-9 specific inhibitor Z-LEHD-fmk, or 10 *μ*M caspase-8 specific inhibitor Z-IETD-fmk for 2 hours before TJ-41 treatment and assessment of apoptosis. All the inhibitors used in this experiment were purchased from Calbiochem San Diego, Calif, USA. 

### 2.5. Preparation of Cytosolic Extracts and Western Blot Analysis of Cytochrome c Release

BrCa cells were washed twice with ice-cold PBS and scraped off the plates. Cells were collected by centrifugation at 500 g for 10 minutes. The cell pellet was resuspended in 500 *μ*L of extraction buffer containing 210 mM mannitol, 70 mM sucrose, 20 mM HEPES-KOH, 50 mM KCL, 5 mM EGTA, 2 mM MgCl2, 1 mM dithiothreitol, 0.1 mM phenylmethylsulfonyl fluoride, and protease inhibitors (Complete Cocktail; Roche Molecular Biochemicals, Indianapolis, Ind). After incubation on ice for 30 minutes, cells were sonicated for 30 seconds. Homogenates were centrifuged at 12.000 × g for 5 minutes at 4°C. Supernatants were collected and further centrifuged at 100.000 × g for 30 minutes at 4°C to yield cytosol. Twenty-five micrograms of total protein, as determined by the Bradford method (Bio-Rad), were resolved on 12% SDS-polyacrylamide gel electrophoresis and transferred to polyvinylidene difluoride (PVDF) membrane. Membrane was blocked with 5% nonfat dry milk in TBS (20 mM Tris-HCL, 8 g/L NaCl, pH 7.4) for 1 hour at room temperature, followed by incubation with 1 *μ*g/mL primary monoclonal anticytochrome c antibody in TBS containing 5% nonfat milk overnight at 4°C. Membrane was washed three times with TBS and blotted with secondary antibody conjugated with horseradish peroxidase (1 : 10.000 dilution, Cell Signaling Technology, Danvers, Mass) at room temperature for 30 minutes. The proteins were visualized using ECLplus (Enhanced Chemiluminescence, Amersham, Piscataway, NJ) after washing three times in TBS.

### 2.6. Western Blotting to Examine Phosphorylation of JNK

After treatments, BrCa cells were washed with ice-cold PBS and lysed with 500 *μ*L of lysis buffer (10 mM Tris-HCL, pH 7.4, 50 mM sodium chloride, 30 mM sodium pyrophosphate, 50 mM sodium fluoride, 100 mM sodium orthovanadate, 2 mM iodoacetic acid, 5 mM zinc chloride, 1 mM phenylmethylsulfonyl fluoride, and 0.5% Trition-×100). The lysates were homogenized by passing through a 22-G needle three times, and kept on ice for 30 minutes. The homogenates were centrifuged at 12 000 g for 15 minutes at 4°C. The protein concentration of whole cell lysates was determined by Bio-Rad protein assay kit. An equal amount of protein (50 *μ*g) was then resolved on a 10% SDS-polyacrylamide gel and transferred to PVDF membrane. The membrane was blocked in 5% nonfat dry milk in TBS for 1 hour at room temperature then incubated overnight at 4°C with 10 *μ*L of anti-phospho-JNK (Thr 183/Tyr185) (1 : 5000 dilution, Cell Signaling Technology). After hybridization with primary antibody, the membrane was washed with TBS for three times, then incubated with horseradish peroxidase-conjugated secondary antibody (1 : 10000 dilution, Cell Signaling Technology) for 30 minutes at room temperature and washed with TBS three times. Final detection was performed with ECL reagents (Amersham, Piscataway, NJ).

### 2.7. Statistical Analysis

All data were analyzed using Sigmastat software (SPSS, Chicago, IL). All treatments were compared with no treatment or 5-FU treated group using one-way ANOVA. If the variance was equal and a significant difference (*P* < .05) was observed, the Holm-Sidak method as a multiple comparison versus control group was used [16]. For data with unequal variance, the Kruskal-Wallis one-way ANOVA on ranks was used. If a significant difference (*P* < .05) was observed, multiple comparisons versus the control group were performed using the Dunn's method.

## 3. Results

### 3.1. Antiproliferative Effect of TJ-41 on BrCa Cell Lines

**Figure 1:**
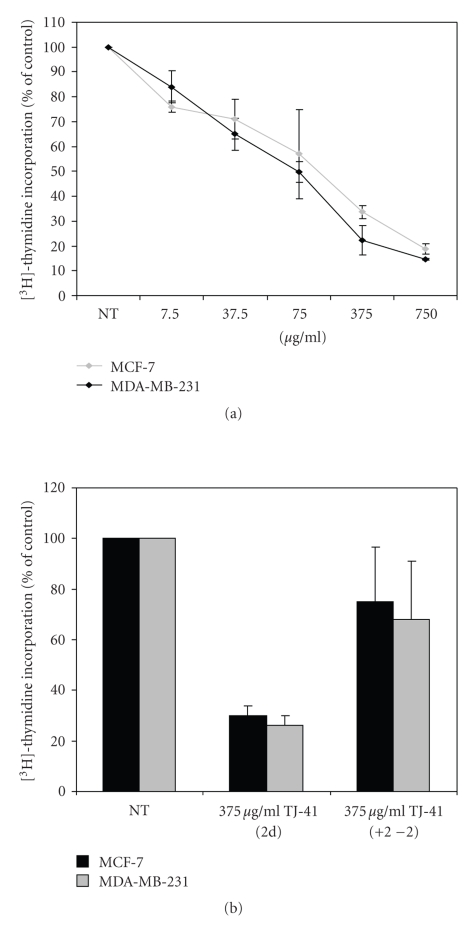
Effect of TJ-41 on the growth of human BrCa cell lines. (a) Two BrCa cell lines (differing in the estrogen receptor status) were cultured in the presence of various concentrations (7.5–750 *μ*g/mL) of TJ-41 for 48 hours. Cell proliferation was measured by ^3^H-Thymidine incorporation in triplicate wells. Results of data derived from three different experiments are expressed as mean percentage of ^3^H-Thymidine incorporation from triplicate wells as compared to that of control (untreated) cells. All data shown are the mean ± SD of three separate experiments. (b) Depicts the reversible effects of TJ-41 on BrCa Cell lines. MCF-7 and MDA-MB-231 cell were cultured in the presence of 375 *μ*g/mL TJ-41 for 2 days. After 2 days, the growth medium was substituted with fresh medium for another 2 days. Cell proliferation was measured by ^3^H-Thymidine incorporation in triplicate wells. Results of data derived from three different experiments are expressed as mean percentage of ^3^H-Thymidine incorporation from triplicate wells as compared to that of control (untreated) cells. All data shown are the mean ± SD of three separate experiments.

 We first investigated the effect of TJ-41 on the growth of estrogen receptor positive (MCF-7) and estrogen receptor negative (MDA-MB-231) breast cancer cell lines in vitro by measuring ^3^H-Thymidine incorporation. As shown in Figure 1(a), exposing these cell lines to TJ-41 for 48 hours induced a dose-dependent decrease in cell proliferation compared with proliferation of untreated cells. TJ-41 at a concentration of 75 *μ*g/mL caused a 50% and 57% growth inhibition in MDA-MB-231 and MCF-7 cells, respectively. We also examined the time-response relationship of TJ-41 on both the cell lines (data not shown). Incubating these cells with media containing TJ-41 for 24, 48, and 72 hours had similar inhibition in cell growth, and the results were not statistically significant.

To further characterize the effects of TJ-41 on BrCa cell lines, we conducted experiments to see if the antiproliferative effects were reversible. When MCF-7 and MDA-MB-231 cells were exposed to 375 *μ*g/mL of TJ-41 for 48 hours, the growth rate was diminished by 72% for MCF-7 (*P* < .05) and 74% for MDA-MB-231 (*P* < .05). Upon removal of the drug for 2 days, the growth rate increased, albeit still at a slower rate for the initial 48 hours (Figure 1(b)). Taken together, these results clearly demonstrate that TJ-41 exerts a dose-dependent antiproliferative effect on BrCa cells and that this response is reversible. Importantly, this antiproliferative effect is ER-independent.

### 3.2. TJ-41 Enhances the Efficacy of 5-Fluorouracil (5-FU)

**Figure 2:**
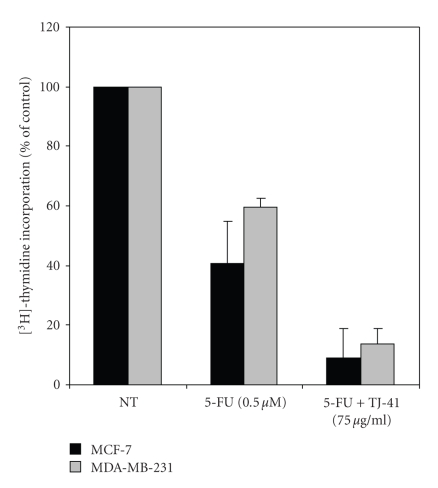
*Effects of TJ-41 on 5 FU-induced cell death on BrCa cell lines*. MCF-7 and MDA-MB-231 cells were treated with 0.5 *μ*M of 5-FU alone and in combination with 75 *μ*g/mL TJ-41 for 48 hours. Cell proliferation was measured by ^3^H-Thymidine incorporation in triplicate wells. Results of data derived from three different experiments are expressed as mean percentage of ^3^H-Thymidine incorporation from triplicate wells as compared to that of control untreated cells. All data shown are the mean ± SD of three separate experiments.

TJ-41 is traditionally prescribed to relieve adverse effects of chemotherapy; therefore, we next conducted experiments to evaluate whether TJ-41 can augment the antiproliferative actions of 5-FU. To determine the cytotoxic activity of 5-FU on MCF-7 and MDA-MB-231 cells, dose-effect experiments were performed (data not shown). For the combined experiments suboptimal doses of 5-FU (0.5 *μ*M) and TJ-41 (75 *μ*g/mL) were used. As shown in Figure 2, treatment of both MCF-7 and MDA-MB-231 cells with 5-FU (0.5 *μ*M) for 48 hours markedly reduced the amount of incorporated [^3^H]-Thymidine by approximately 41% for MCF-7 (*P* < .001) and 59% for MDA-MB-231 (*P* < .001). When cells were cotreated with 5-FU and TJ-41 they measured 51% and 73% (*P* < .001) in MCF-7 and MDA-MB-231, respectively. These results indicated that TJ-41 may enhance the antiproliferative ability of 5-FU in both ER+ and ER− BrCa cell lines. 

### 3.3. TJ-41 Induces Apoptosis and Potentiates the Apoptotic Effects of 5-FU

**Figure 3:**
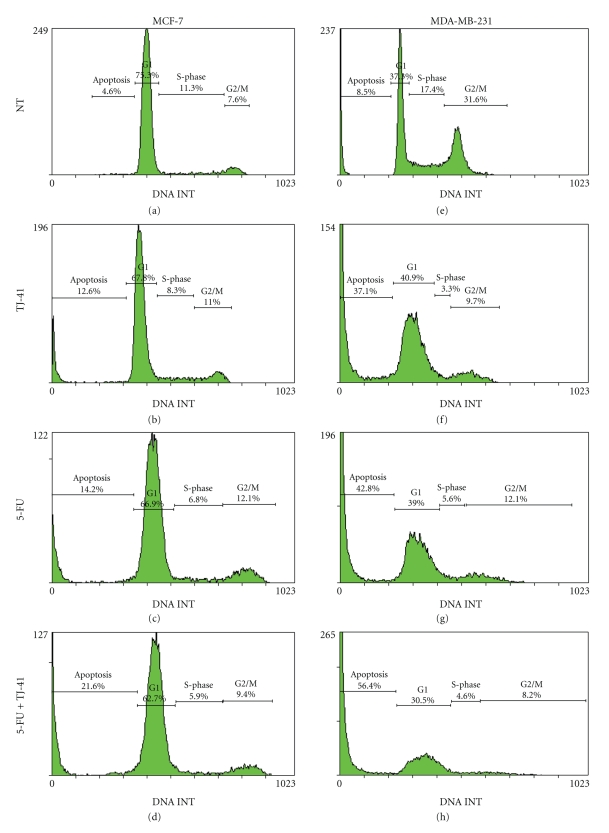
*Cell cycle kinetics of BrCa cells after treatment with TJ-41 and 5-FU*. MCF-7 and MDA-MB-231 cells were treated with TJ-41 and 5-FU either alone or combined for 48 hours. DNA was stained with propidium iodide. The figure shows the cell cycle profile of control cells (a) and (e), TJ-41-treated cells (b) and (f), 5-FU treated cell (c) and (g), and TJ-41 and 5-FU combined (d) and (h). Results are from one experiment that is representative of three similar ones.

**Figure 4:**
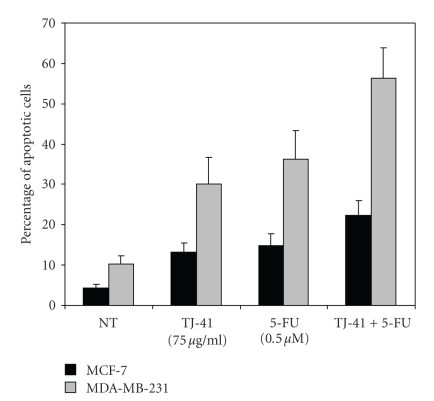
The effect of TJ-41 and 5-FU treatment on apoptosis (sub G_1_) of MCF-7 and MDA-MB-231 cells measured after 48 hours by flow cytometry. Percentages of apoptosis were calculated from flow cytometry measurements of PI-stained DNA as described in Methods and Materials. Data represent mean ± SD of at least three experiments.

To determine the mechanism of TJ-41 induced growth inhibition, we studied the effects of test compounds with respect to the induction of apoptosis in the BrCa cells. MCF-7 and MDA-MB-231 cells were grown in twelve-well plates and were treated with TJ-41 and 5-FU alone and in combination for 48 hours. Cell death was assayed by propidium iodide staining and subjected to flow cytometric analysis. As shown in Figure 3, treatment of MCF-7 and MDA-MB-231 cells with TJ-41 and 5-FU led to a cell cycle phase distribution with a decrease of cells in S-phase associated with a concomitant increase of the cell portion predominantly in sub-G_1_ phase (Figures 3(b)– 3(g)). Again, in both of the cell lines, combination of TJ-41 and 5-FU caused a significant increase in the sub-G_1_ cell population (Figures 3(d)  and 3(h)) compared to no treatment (Figures 3(a)  and 3(e)). A histogram summarizing the percentage of sub-G_1 _fraction of cells is shown in Figure 4. For both BrCa cell lines, cotreatment of TJ-41 and 5-FU led to a greater increase in the percentage of sub-G_1_ cell population than either one alone. TJ-41 (75 *μ*g/mL) treatment produced a significant increase in the percentage of the sub-G_1_ cell population (control cells: 4 ± 1% for MCF-7 and 10 ± 2% for MDA-MB-231; TJ-41 : 13 ± 9% for MCF-7 and 31 ± 5% for MDA-MB-231). Similarly, the 5-FU treatment also produced a significant elevation of the sub-G_1_ cell population (control cells: 4 ± 1% for MCF-7 and 10 ± 2% for MDA-MB-231; 5-FU: 15 ± 3% for MCF-7 and 36 ± 7% for MDA-MB-231). When TJ-41 and 5-FU were combined, the increase in the sub-G_1_ cell population was much more pronounced (control cells: 4 ± 1% for MCF-7 and 10 ± 2% for MDA-MB-231; TJ-41 + 5-FU: 22 ± 4% for MCF-7 and 56 ± 8% for MDA-MB-231). 

### 3.4. TJ-41 Induced the Release of Cytochrome c

**Figure 5:**
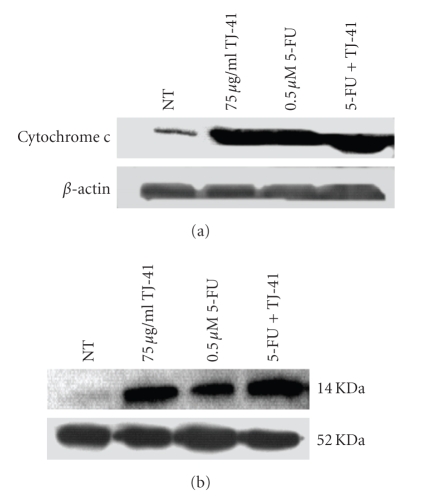
*Effect of TJ-41 on the release of cytochrome c from mitochondria*. After treatment cytosolic extracts of BrCa cell lines were prepared as described in the Materials and Methods. Twenty-five micrograms of cytosolic protein were resolved on 12% SDS-polyacrylamide gel electrophoresis and transferred to PVDF membrane. Membrane was blocked with 5% nonfat dry milk in TBS, followed by incubation with 1 *μ*g/mL primary monoclonal anticytochrome c antibody in TBS containing 5% nonfat milk overnight at 4°C. The proteins were visualized using analyzed by western blotting using anticytochrome c antibody.

To examine the possible role of mitochondria in the apoptotic death of MCF-7 and MDA-MB-231 cells induced by TJ-41, the existence of cytochrome c in the cytosolic fraction of cell lysis was measured. Treatment of cells for 48 hours with 75 *μ*g/mL TJ-41 led to a dramatically increased amount of cytochrome c leaking from mitochondrial membrane into cytosol in both MCF-7 (Figure 5(a)) and MDA-MB-231 (Figure 5(b)) cells. Consistent with the flow cytometric analysis, combined treatment of 5-FU and TJ-41 led to a larger release of cytochrome c.

### 3.5. TJ-41 Induced JNK Activation and Inhibition of JNK and Caspase-9 Abrogates TJ-41-Induced Apoptosis

**Figure 6:**
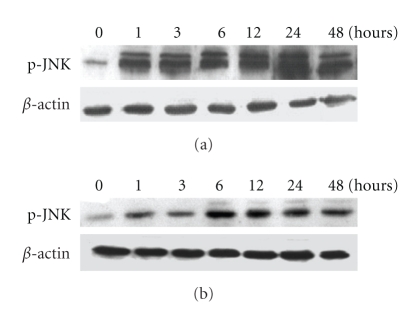
*Effect TJ-41 induced JNK activation*. (a) MCF-7 and (b) MDA-MB-231 cells were treated with 75 *μ*g/mL TJ-41 and cell lysates were collected at the indicated times. Total cellular proteins were isolated and Wstern blot were performed as described in the Materials and Method. All samples in gels are equally loaded at 50 *μ*g of total protein, and *β*-actin was used as an internal control for equal protein loading.

 Since the JNK pathway has been demonstrated to be required for apoptosis caused by chemotherapeutic agents [17], it is conceivable that the JNK pathway might also play a key role in TJ-41-induced apoptosis in BrCa cells. To test this, we performed Western blot analysis to examine phosphorylation of JNK (p-JNK) protein. The p-JNK protein expression was detectable as early as 1 hour after TJ-41 treatment in both cell lines; however, the activation peaked at ~24 hours for MCF-7 cells and 6 hours for MDA-MB-231 cells (Figure 6). 

**Figure 7:**
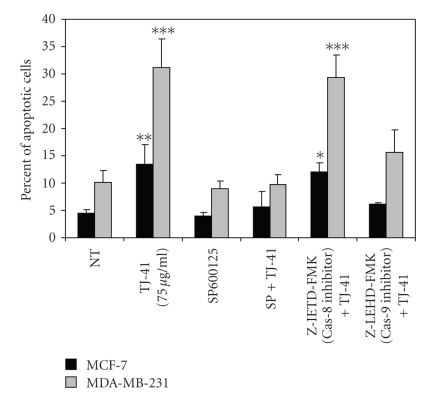
*Effects of SP600125 (a JNK inhibitor), Z-IETD-FMK (a caspase-8 inhibitor), and Z-LEHD-FMK (a caspase-9 inhibitor) on TJ-41-induced apoptosis in MCF-7 and MDA-MB-231 breast cancer cell lines*. BrCa cells were exposed to inhibitors for one hour prior to the addition of TJ-41 (48 hours). Percent of cells undergoing apoptosis (sub G_1_) was measured by flow cytometry of propidium-iodide stained DNA. Data represents mean ± SD of at least three experiments. Significant differences from untreated control are indicated: *P < .05, **P < .002, and ***P < .001.

 To further elucidate the apoptotic signaling pathway triggered by TJ-41, we investigated the effects of SP600125 (a JNK inhibitor), Z-IETD-FMK (a caspase-8 inhibitor), and Z-LEHD-FMK (a caspase-9 inhibitor) on TJ-41 induced cell death. Cells were analyzed by flow cytometry, and the extent of apoptosis was determined by measuring the fraction of cells with sub-G_1_ DNA content. As shown in Figure 7, treatment of BrCa cells with the JNK inhibitor SP600125 alone for 1 hour had negligible effects on apoptosis compared to the no treatment group. However, when cells were treated with a combination of SP600125 (1 hour) and TJ-41 (47 hours), the percentage of cells undergoing apoptosis was dramatically lower than those with TJ-41alone, indicating that inhibition of JNK activation protects against the apoptotic effects of TJ-41. Similarly, combined treatment of cells with the caspase-9 inhibitor Z-LEHD-FMK and TJ-41 led to marked decrease in the percent of cell undergoing apoptosis. However, in the presence of caspase-8 inhibitor Z-IETD-FMK, TJ-41's proapoptotic effect was not affected. Thus the activation of JNK and caspase-9 but not caspase-8 is necessary and sufficient for the induction of apoptosis in response to TJ-41 treatment.

## 4. Discussion and Conclusion

 Breast cancer causes significant morbidity and mortality and is a major public problem in the United States and increasingly in the developing world. Conventional chemotherapeutic drugs such as 5-Fluorouracil (5-FU) are widely used in cancer therapy, but the nonspecificity and acquired drug resistance as well as induction of side effects due to high dosage chemoresistance remains a major obstacle in clinical settings. Therefore, much attention has been focused on natural products as potential sources of novel anticancer drugs over the last few decades [18]. With an annual sale value of $52 million in Japan alone, TJ-41 is heavily prescribed to treat such conditions as general fatigue and poor appetite to help promoting overall physical recovery following operations. By nourishing the blood and toning the gastrointestinal (GI) system, administration of TJ-41 has been reported to be able to supplement or relieve the adverse events of cancer chemotherapy [19]. Although the major uses of TJ-41 are to relieve stress and fatigue, boost immunity, and speed up the recovery of total white-blood-cell counts, this tonic formula may also have additional chemopreventive or therapeutic effects on breast cancer. Here, we report that TJ-41 can effectively inhibit hormone sensitive and insensitive breast cancer cell lines (MCF-7 and MDA-MB-231, resp.) and enhance the efficacy of 5-FU.

 Invasive breast cancers can be divided into two subtypes based on whether or not the tumor cells express estrogen receptor (ER). The ER status is important because, when circulating estrogen binds ER, it stimulates cell division and tumor growth [20]. Many strategies have been developed over the years to inhibit this estrogen-induced mitogenic pathway. Numerous studies have shown that hormonal therapies significantly prolong life, although they rarely cure patients with ER-positive (ER+) breast cancer, where the mitogenic pathway is intact, but that they are ineffective in ER-negative (ER−) disease, where the pathway is inactive [20]. There are three general goals for better therapies in breast cancer, improving therapies for ER+ disease, finding therapies for ER− disease and, especially, broadening therapies for prevention to include ER− disease. In the present study, our results showed that the water-soluble ingredients of TJ-41, at a concentration of 75 *μ*g/mL, displayed antiproliferative effects on both MCF-7 (ER+) and MDA-MB-231 (ER−) breast cancer cell lines. Importantly, this antiproliferative effect was reversible and independent of the estrogen receptor status. The concentrations of TJ-41 used in our experiments are the same as the blood concentrations attained when 7.5 g (the recommended dose) of TJ-41 is administered orally. Among the components of TJ-41, cimicifuga rhizome (BrCa), glycyrrhiza root (BrCa), and Ginseng were reported to suppress the proliferation of breast cell lines in vitro [21–23]. It is therefore reasonable to assume that the direct inhibitory effect of TJ-41 on the breast cancer cell lines shown in our study may be caused by those active ingredients and perhaps additional as yet unidentified ingredients. 

Recently, the pharmacological manipulation of growth inhibition and antiproliferative effect of malignant cells through the induction of apoptosis have been recognized as a novel strategy for the identification and screening of potential chemotherapeutic agents. Many chemotherapeutic agents have been found to retain the activity of apoptosis [25, 26]. The present study reveals that the growth inhibiting effects of TJ-41 appeared to be associated with the induction of apoptosis (Figure 3). The TJ-41-induced apoptosis was confirmed by flow cytometric analysis (Figures 3  and 4).

There are two pathways currently proposed to play major roles in regulating apoptosis in mammalian cells: an extrinsic pathway mediated by one or more death receptors and an intrinsic pathway mediated by mitochondria [27]. In the extrinsic death receptor/ligands pathway, caspase activation occurs as a direct consequence of death receptor ligation, with upstream caspase-8 cleaving and activating downstream proteases such as caspase-9 and caspase-3. In the intrinsic mitochondrial pathway, Bax, a member of the Bcl-2 family, plays the leading role. Bax normally resides in the cytosol in a quiescent state. After an apoptotic stimulus, Bax is translocated into the mitochondria and promotes the release of cytochrome c [28], possibly by forming a pore [29] or a voltage-dependent anion channel in the outer mitochondrial membrane. Once in the cytosol, cytochrome c activates Apaf-1, which then activates procaspase-9, which, in turn, activates caspase-3, triggering apoptosis. The current study found that TJ-41 caused the release of mitochondrial cytochrome c in both cell lines. The fact that TJ-41-induced cell death was reduced inhibitors specific for caspase-9 (Z-LEHD-fmk), but that cell death was not reduced by exposure to Z-IETD-fmk, an inhibitor specific for caspase-8, strongly suggests that the intrinsic mitochondrial pathway is engaged in TJ-41-induced apoptosis.

Activation of the JNK pathway has been shown to be a common phenomenon in apoptotic cell death [30–33]; however, the importance of this activation seems to vary in apoptosis caused by different agents. The JNK pathway is required for apoptosis induction by growth factor withdrawal, heat shock, radiation, and ceramide [32, 34, 35]. In contrast, JNK may not be essential for receptor-mediated apoptosis (e.g., Fas- and tumor necrosis factor-mediated apoptosis) [36]. In this study, we demonstrate the involvement of JNK in TJ-41-induced apoptosis by showing that interfering with the JNK pathway suppressed TJ-41-induced cell death. This result indicates that induction of JNK activity is not a general event caused by stress during cell death, but rather it is a specific phenomenon associated with apoptotic cell death.

5-FU is the most active and widely used chemotherapeutic agent for breast cancer, with a response rate of 40% in previously untreated patients [37]. It intercalates between DNA base pairs, induces DNA strand breaks, and inhibits DNA biosynthesis and topoisomerase II activity [38]. It also forms free radicals [39], which contribute to its cytotoxicity and substantial antitumor activity [40, 41]. However, in majority of the cases, 5-FU does not alleviate breast cancer and is linked with significant physiological and psychological side effects [42]. Additionally, the development of drug-resistant phenotypes remains a significant limitation to its clinical use. The finding of natural inducers of apoptosis will be the basis for the development of chemopreventive potential natural materials [43]. Dietary constituents are known to have a potential in chemoprevention and to be generally safe. A new facet of chemoprevention research is determining whether preventative agents can make chemotherapy work better. This study has examined the molecular evidence to prove chemotherapeutic efficacy of the combination treatment of anticancer agents and natural compounds in order to develop the modalities to overcome drug resistance and cytotoxic effects of breast cancer treatment. Our study demonstrates that TJ-41 enhances the susceptibility of MCF-7 and MDA-MB-231 cells to 5-FU induced apoptosis. This increase in apoptosis, compared to TJ-41 or 5-FU alone, was confirmed by flow cytometric analysis as well as an increase in caspase-3/7 activity (data not shown). The present study for the first time proposes that TJ-41 could be effectively used in combination with 5-FU. 

**Figure 8:**
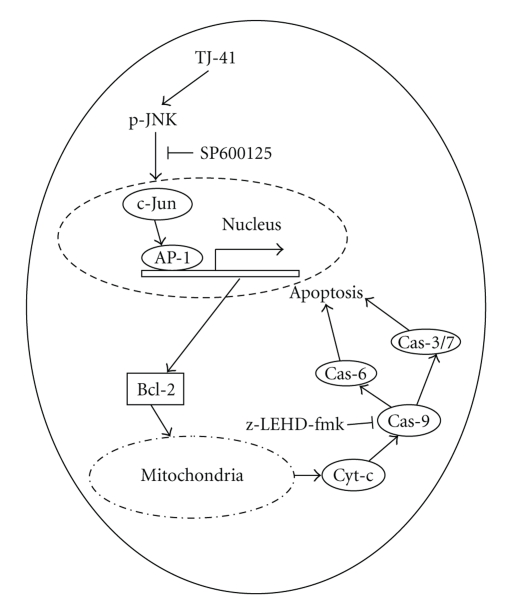
*Proposed schema of molecular events involved in TJ-41 induced apoptosis in MCF-7 and MDA-MB-231 cell lines*. Exposing breast cancer cells to TJ-41 leads to environmental stress thus triggers the activation of JNK. We speculate that phosphorylation (activation) of JNK activates c-Jun which leads to the transcription of proapoptotic Bcl-2 genes. The resulting Bcl-2 proteins are translocated into the mitochondria and promotes the release of cytochrome c, possibly by forming a pore or a voltage-dependent anion channel [24] in the outer mitochondrial membrane. Once in the cytosol, cytochrome c activates procaspase-9, which, in turn, activates other “executioner” caspases such as caspase-3,- 6, and- 7, as a result, committing cell to under going apoptosis.

 In conclusion, we have demonstrated, for the first time that TJ-41, an Asian herbal mixture, exhibits a direct inhibitory effect on human breast cancer cell in vitro. The inhibitory effect can be attributed to the ability of TJ-41 to induce apoptosis. From our results we deduced that exposing TJ-41 to breast cancer cells leads to environmental stress with subsequent activation of JNK which in turn activates apoptosis via the mitochondrial pathway (Figure 8). Additionally, combination of TJ-41 with 5-FU suggests potential therapeutic applications in breast cancer. Further investigations on its possible clinical applications in breast cancer therapy should be carried out, especially in regards to improving chemotherapeutic outcomes of ER− breast cancers.
